# Human granulocytic anaplasmosis in Kinmen, an offshore island of Taiwan

**DOI:** 10.1371/journal.pntd.0007728

**Published:** 2019-09-20

**Authors:** Kun-Hsien Tsai, Lo-Hsuan Chung, Chia-Hao Chien, Yu-Jung Tung, Hsin-Yi Wei, Tsai-Ying Yen, Pei-Yun Shu, Hsi-Chieh Wang

**Affiliations:** 1 Institute of Environmental and Occupational Health Sciences, College of Public Health, National Taiwan University, Taipei, Taiwan; 2 Department of Public Health, College of Public Health, National Taiwan University, Taipei, Taiwan; 3 Center for Diagnostics and Vaccine Development, Centers for Disease Control, Ministry of Health and Welfare, Taipei, Taiwan; 4 Kinmen Hospital, Ministry of Health and Welfare, Kinmen, Taiwan; 5 Taipei Regional Center, Centers for Disease Control, Ministry of Health and Welfare, Taipei, Taiwan; Instituto de Pesquisas Veterinarias Desiderio Finamor, BRAZIL

## Abstract

**Background:**

Human granulocytic anaplasmosis, a tick-borne infection caused by *Anaplasma phagocytophilum*, has received scant attention, while scrub typhus, a mite-transmitted disease caused by *Orientia tsutsugamushi*, is the most common rickettsiosis in Taiwan. The clinical presentations of both diseases are characterized by undifferentiated fever, headache and malaise. Moreover, both pathogens have been detected in small mammals that serve as hosts for chiggers and ticks in the wild. The objective of the present study was to investigate whether human granulocytic anaplasmosis occurs in Taiwan.

**Methodology/Principal findings:**

Blood samples from 274 patients suspected of having scrub typhus in Kinmen, an offshore island of Taiwan, in 2011 and 2012 were retrospectively examined by immunofluorescence assays. IgG antibodies reactive with *Anaplasma phagocytophilum* was found in 31.8% (87/274) of the patients. Paired serology identified 3 patients with human granulocytic anaplasmosis and 8 patients with coinfection with *O*. *tsutsugamushi* and *A*. *phagocytophilum*. Laboratory tests showed that elevated serum ALT/AST, creatinine, and BUN levels were observed in patients with anaplasmosis and coinfection, but elevated serum CRP levels, thrombocytopenia, and anemia were only observed in coinfected patients. PCR detected *A*. *phagocytophilum* 16S rDNA and *p44/msp2* in 2 patients. The phylogenetic analysis suggested that the replicons of the 16S rDNA shared high sequence similarity with the reference sequences in the Korea, USA, Japan, and China. The amplicons of *p44/msp2* were close to those of the human variants identified in the USA and Japan.

**Conclusions:**

Our findings indicated that *A*. *phagocytophilum* infection was prevalent but unrecognized in Taiwan.

## Author summary

Human granulocytic anaplasmosis is a tick-borne rickettsial infection caused by *Anaplasma phagocytophilum*. Although most cases resolve readily, life-threatening complications can occur without prompt antibiotic treatment. The major difficulty in diagnosing human granulocytic anaplasmosis is due to the nonspecific nature of the symptoms. Given that scrub typhus is the most frequently reported rickettsial disease in Taiwan and shares similar early clinical signs with anaplasmosis, we retrospectively examined blood samples from patients with suspected diagnoses of scrub typhus in 2011 and 2012. While serological evidence of potential past exposure was found in as many as 31.8% (87/274) of the patients, current or recent anaplasmosis was supported by seroconversion in 11 patients, including 8 patients coinfected with scrub typhus. *Anaplasma phagocytophilum* DNA was detected in acute phase samples, and the amplified fragments were phylogenetically close to those of variants in the Korea, the USA, Japan, and China. Herein, for the first time, we confirmed the presence of human granulocytic anaplasmosis in Taiwan. By reporting coinfections with anaplasmosis and scrub typhus, the study further highlighted the health risk of increasing contact with wild rodents.

## Introduction

Human granulocytic anaplasmosis (HGA) is an emerging rickettsial disease caused by *Anaplasma phagocytophilum*. Since it was first identified in the United States, HGA has been reported across Europe and in China, Japan, and South Korea [1–12]. The disease is transmitted by *Ixodes* ticks, although the species varies according to the habitat, with *Ixodes scapularis* and *Ixodes pacificus* found in North America, *Ixodes ricinus* found in Europe, and *Ixodes persulcatus* found in Asia [10, 11, 13]. Other genera, such as *Dermacentor* spp. and *Rhipicephalus* spp. have been reported to be biological vectors, but their significance remains unknown [14, 15]. Larval or nymphal ticks acquire the bacterium via feeding on infected small mammals before transferring it to humans or domestic animals during their subsequent life stages. Small mammals, including white-footed mice (*Peromyscus leucopus*), woodrats, squirrels (*Sciurus* spp.), chipmunks (*Tamias* spp.), voles, hedgehogs, and shrews are known reservoirs for the rickettsial pathogen [16].

*Anaplasma phagocytophilum* is an obligate intracellular, Gram-negative bacterium which attacks granulocytes, neutrophils especially. The bacterium enters the host cell by phagocytosis via binding between the fucosylated or sialylated scaffold proteins, e.g. PSGL-1 (CD162) and L-selectin, on the granulocyte surfaces and the bacterium surface protein, e.g. p44/Msp2 [17, 18]. It has been reported that infection changes gene expressions that modify endocytic pathway and prolong the life of host granulocytes [19, 20]. The pathogen then replicates by binary fission in an endosome, growing into a cluster called morulae until being released by exocytosis or apoptosis of the host cell. Individuals who have contracted HGA often present with fever, malaise, myalgia, and headache [21]. Although most patients recover spontaneously in a short period of time, as with other rickettsial infections, poor outcomes can occur without prompt treatment. Approximately one-third to one-half of symptomatic patients require hospitalization, and 3% to 7% develop life-threatening complications, with fatality rates less than 1% [22]. HGA can be difficult to diagnose because of the nonspecific nature of the symptoms, but antibiotic therapy needs to be administered as early as possible in the course of the illness when it is most likely to be successful. Doxycycline is the first-line treatment for anaplasmosis in adults and children. Therapy for a presumptive diagnosis should be initiated while waiting for laboratory confirmation via serologic tests, the detection of bacterial DNA by PCR, or bacterium isolation by culturing [1].

In Taiwan, human cases of granulocytic anaplasmosis have not been formally reported, but *A*. *phagocytophilum* infections have been identified in *Rattus losea*, *Rattus norvegicus*, *Mus caroli*, dogs, and one nymph each of *Ixodes granulatus* and *Rhipicephalus haemaphysaloides*, implying that the pathogen is being transmitted [23–27]. Scrub typhus, in contrast, is listed as a notifiable disease along with epidemic typhus and murine typhus, and it is the best recognized rickettsial disease. Transmitted by trombiculid mites, *Orientia* bacteria multiply in the inoculation site and disseminate into multiple organs through endothelial cells and macrophages, resulting in the development of fatal complications [28]. The incidence rate of scrub typhus was 1.9 per 100,000 person-years from 2008 to 2017 while certain offshore island such as Kinmen had an incidence rate as high as 51.6 per 100,000 person-years, but only 13.1–19.9% of the blood samples collected for laboratory diagnosis actually tested positive for *Orientia* infection [29]. The etiological agents of a rather large proportion of rickettsia-like fevers remained to be determined; hence, the current retrospective study was conducted to investigate whether HGA is present in Taiwan.

## Methods

### Ethics statement

The use of samples and medical records was approved by the Institutional Review Board of the Taiwan Centers for Disease Control (Taiwan CDC) (No. 102006) and the National Taiwan University Hospital Research Ethics Committee (No. 201806011RIND). Blood samples from patients with suspected scrub typhus were sent to the Taiwan CDC laboratory for diagnosis as routine practice. Further application of the leftover specimens was approved by a written informed consent. The material transfer agreement for the samples was officially granted by the Taiwan CDC (No. 1070001530). All data analyzed were anonymized.

### Study sites and blood samples

Kinmen County consists of a group of offshore islands governed by Taiwan and is located approximately 2 kilometers away from mainland China. Remaining a military reserve until the mid-1990s, development on the islands has been limited. A quarter of the area of the county has been designated as a national park which is famous for migratory birds and wildlife. Human population continuously grew during the past decade, from 84,570 in 2008 to 137,456 in 2017. It is one of the counties with the highest prevalence of scrub typhus in Taiwan.

Kinmen Hospital is the only regional and referral hospital in Kinmen County. Blood samples from 274 patients presenting with clinical symptoms resembling those of scrub typhus were sent to the Taiwan CDC for laboratory diagnosis from 2011 to 2012 (8–72 years of age, mean 26.2 years). *Orientia* infection was diagnosed when one of the following criteria was met: (1) the isolation of *O*. *tsutsugamushi* from blood or eschars, (2) the detection of *O*. *tsutsugamushi* DNA, (3) total antibody titers for IgM≥1:80 and IgG≥1:320, or (4) a ≥4-fold increase in antibody titers in paired sera.

### Immunofluorescence assay (IFA)

Infection of *A*. *phagocytophilum* was examined by immunofluorescence assay (IFA) using the Focus *Anaplasma phagocytophilum* (HGA) IFA IgG Kit (Focus Technologies, Cypress, CA, USA). Patients’ serum samples were diluted from 1:64 to 1:2048, and the reaction was read at a final magnification of 400X under a fluorescence microscope (Leica Microsystems, Singapore). An IgG endpoint titer ≥1:64 was suggestive of exposure according to the manufacturer’s instructions. A ≥4-fold increase in antibody titers in paired sera indicated current or recent infection.

Scrub typhus was diagnosed by an in-house IFA [30]. The serum samples were diluted from 1:40 to 1:640 and reacted with *O*. *tsutsugamushi* (Karp + Kato + Gilliam strains)-infected L929 cells coated on the slides. The reactive antibodies were detected with FITC-conjugated secondary antibodies, and the slides were then observed under a fluorescence microscope.

### Clinical manifestations and characteristics of HGA cases

The medical records of patients with HGA were reviewed retrospectively. The demographic information, clinical manifestations, the results of laboratory tests, clinical diagnoses, comorbidities, and antimicrobial treatments were recorded. The geographic distribution of the patients was mapped manually using the Microsoft Paint and a background map available on USGS LandsatLook (https://landsatlook.usgs.gov/) according to their residential addresses.

### Molecular diagnosis

DNA was extracted from the blood and buffy coats using a QIAamp DNA Blood Mini Kit (QIAGEN, Hilden, Germany). PCR was performed using the primers EHR16SD (5’-GGTACCYACAGAAGAAGTCC-3’) and EHR16SR (5’-TAGCACTCATCGTTTACAGC-3’), which amplify a 345-bp fragment of the 16S rDNA of the Anaplasmataceae family [31]. The reaction was run on a Biometra TRIO thermocycler (Analytik Jena AG, Jena, Germany) with the following conditions: 94°C for 15 min, 35 cycles of 94°C for 30 s, 53°C for 30 s, and 72°C for 1 min, followed by termination at 72°C for 10 min. Infection with *A*. *phagocytophilum* was further assessed by nested PCR targeting the multiple-copy *p44/msp2* gene as previously described [10]. The set of external primers p3726 (5’-GCTAAGGAGTTAGCTTATGA-3’) and p4257 (5'-AAGAAGATCATAACAAGCATT-3’) and the set of internal primers p3761 (5’-CTGCTCTKGCCAARACCTC-3’) and p4183 (5’-CAATAGTYTTAGCTAGTAACC-3’) were used for amplification. The reaction conditions were 94°C for 15 min, 35 cycles of 94°C for 30 s, 52°C for 30 s, and 72°C for 1 min, followed by 72°C for 10 min. For all reactions, negative water controls were included during each run. The *p44*/*msp2* amplicons from positive samples were then cloned into a pCR2.1 vector with the TA Cloning Kit (Life Technologies, Grand Island, NY, USA). For scrub typhus, real-time PCR was also used to detect the 56-kDa type-specific antigen (TSA) gene [32]. The reaction was run on an iQ5 iCycler (BioRad Laboratories, Hercules, CA, USA) using the KAPA SYBR FAST Universal Kit (Sigma-Aldrich Corporation, St. Louis, MO, USA) following the manufacturer’s instructions. Samples were considered positive if they had a cycle threshold value <50 and characteristic amplification plots.

The PCR products generated in the study were sent for sequencing in both the forward and reverse directions (Mission Biotech, Taipei, Taiwan). Sequences were aligned using SeqMan Pro (Lasergene, Madison, USA) and evaluated for homology with previously reported sequences by a BLAST search of the GenBank database (http://blast.ncbi.nlm.nih.gov/Blast.cgi). A phylogenetic tree was constructed based on the alignment and the most closely related paralogs, followed by the application of Maximum Likelihood method or Neighbor-Joining method (1,000 bootstrap) using MEGA7 software [33].

### Statistical analysis

All statistical analyses were performed with SAS v9.1.3 (SAS Institute, Cary, NC). Categorical variables were compared with Chi-square tests, and continuous variables were analyzed with t-tests; p≤0.05 was considered statistically significant.

### Accession numbers

Sequences generated in the study have been uploaded to GenBank.

*Anaplasma phagocytophilum* 16S rDNA: MH260385, MH260386, MH260387, MH260388, MH260389, MH260390, MH260391, MH260392.

*Anaplasma phagocytophilum p44/msp2*: MH260370, MH260371, MH260372, MH260373, MH260374, MH260375.

## Results

### *Anaplasma phagocytophilum* infection

Of the 274 patients suspected of having scrub typhus, 129 cases (129/274; 47.1%) were confirmed by the Taiwan CDC laboratory. Moreover, 87 were positive for *A*. *phagocytophilum*-specific IgG (87/274; 31.8%) (Table 1). There were no significant differences in positivity rates according to gender, occupation, or age group. Four-fold increases in *A*. *phagocytophilum* IgG titers were observed in 11 paired serum samples (patients A-K) (Table 2). While 3 of those patients appeared to have only HGA (patients A-C), 8 of the patients also showed seroconversion against *O*. *tsutsugamushi*, suggesting coinfection (patients D-K).

**Table 1:** Seroprevalence of *Anaplasma phagocytophilum* in patients suspected of having scrub typhus from Kinmen County, 2011–2012.

	Sample No.	Seropositive No.	Seropositive rate (%)	*p* value
**Gender**				
Male	208	67	32.2	0.77
Female	66	20	30.3	
**Occupation**				
Military service	57	15	26.3	0.55
Agriculture, forestry, fishing, animal husbandry	22	8	36.4	
Housekeeping, student	101	30	29.7	
Business	63	21	33.3	
Public service	31	13	41.9	
**Age**				
>70	39	12	30.8	0.20
60–69	42	10	23.8	
50–59	51	21	41.2	
40–49	31	9	29.0	
30–39	33	12	36.4	
20–29	55	15	27.3	
10–19	18	7	38.9	
<10	5	1	20.0	
**Total**	274	87		

**Table 2:** Results of the serological and molecular analyses of the 11 patients with human granulocytic anaplasmosis (HGA) and scrub typhus in Kinmen County, 2011–2012.

Disease	Patients	1˚ sampling (days after onset)	2˚ sampling (days after onset)	*Anaplasma phagocytophilum*				*Orientia tsutsugamushi*				
				IFA (IgG) (X^-1^)		PCR		IFA (IgM) (X^-1^)		IFA (IgG) (X^-1^)		PCR
				Acute phase	Convalescent phase	16S rDNA	*p44*/*msp2*	Acute phase	Convalescent phase	Acute phase	Convalescent phase	56kDa TSA gene
HGA	A	1	21	256	≥ 2048	+	−	−	−	−	−	−
	B	2	16	256	1024	+	−	−	−	−	−	−
	C	NA	NA	128	512	+	−	−	−	−	−	−
Co-infection of HGA and scrub typhus	D	6	16	128	1024	+	+	40*	160	40*	640	+
	E	6	16	−	512	+	+	−	160	−	320	+
	F	4	12	128	512	+	−	40	80	80	≥ 640	+
	G	2	23	−	256	+	−	≥ 160	NA	≥ 640	NA	+
	H	NA	NA	64	256	+	−	−	80	−	≥ 640	−
	I	1	15	64	≥ 2048	−	−	40*	≥ 160	40*	≥ 640	−
	J	3	18	128	512	−	−	−	80	−	320	−
	K	1	18	−	512	−	−	−	160	−	640	−

+: positive; −: negative

*: screened with FITC-IgG/A/M (1:40); NA: not available; TSA: Type specific antigen.

### Clinical manifestations and characteristics of HGA cases

**Fig 1:**
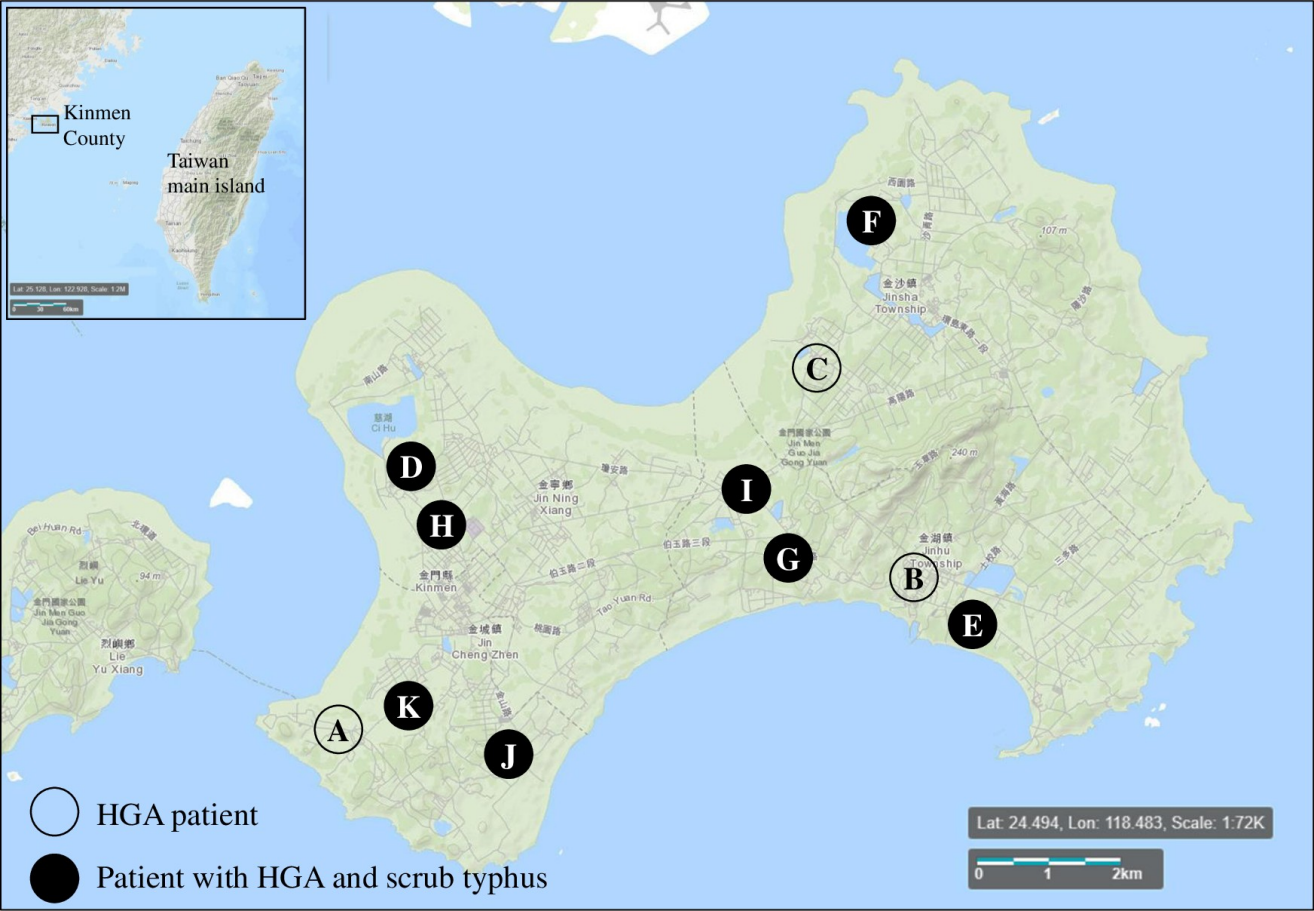
Distribution of the patients with human granulocytic anaplasmosis (HGA) in the study. The geographic distribution of the patients was mapped manually using the Microsoft Paint and a background map available on USGS LandsatLook (https://landsatlook.usgs.gov/) according to their residential addresses.

The complete medical records of 9 HGA patients (patients A, B, D-G, I-K) were retrieved from Kinmen Hospital and carefully reviewed. These patients lived in different villages on the island (Fig 1), and the infections mostly occurred in June (n = 6) and July (n = 3) when scrub typhus peaked in the years (S1 Fig). The symptoms were summarized in Table 3. All patients developed fever (9/9), while eschars at a variety of sites (knee, axillary area, back and inguinal area) were only found in patients coinfected with *O*. *tsutsugamushi*. Laboratory tests showed that elevated serum ALT/AST, creatinine, and BUN levels were observed in patients with *A*. *phagocytophilum* infection, but elevated serum CRP levels, thrombocytopenia, and anemia were only observed in patients with concurrent scrub typhus and HGA. HGA/scrub typhus coinfection did not seem to negatively impact on the clinical outcomes of patients. All patients recovered after treatment with minocycline or doxycycline (oral or intravenous administration). With regard to the patients’ contact and travel histories, one of the HGA patients (patient B) returned from a trip to Guangxi Province in China a week before the onset of symptoms; one patient (patient A) had traveled to Taiwu mountain, and another patient (patient I) had a history of contact with cattle. However, all patients denied having experienced a recent tick bite (S1 Table).

**Table 3:** Clinical symptoms of the 9 patients with human granulocytic anaplasmosis (HGA) in Kinmen County, 2011–2012.

**Clinical symptoms**	**Patients, n/N**		**Laboratory findings**	**Patients, n/N**	
	Coinfection of HGA and scrub typhus	Infection of HGA		Coinfection of HGA and scrub typhus	Infection of HGA
Fever	7/7	2/2	Elevated CRP	5/5	0/1
Eschars	5/7	0/2	Elevated ALT	6/7	1/2
Malaise	3/7	0/2	Elevated AST	5/6	1/2
Chills	2/7	1/2	Elevated LDH	2/3	0/0
Cough	1/7	1/2	Elevated Creatinine	4/6	1/2
Headache	1/7	1/2	Elevated BUN	2/4	1/1
Poor appetite	1/7	1/2	Thrombocytopenia	3/7	0/2
Rash	1/7	1/2	Anemia	2/7	0/2
Abdominal discomfort	1/7	0/2	**Antibiotics treatments**	**Patients, n/N**	
Diarrhea	1/7	0/2		Coinfection of HGA and scrub typhus	Infection of HGA
			Minocycline only	6/7	0/2
Nausea	1/7	0/2	Doxycycline only	0/7	2/2
Sore throat	1/7	0/2	Minocycline + Doxycycline	1/7	0/2
Sputum	0/7	1/2			
Syncope	1/7	0/2			
Vomit	1/7	0/2			
Body aches and muscle pain	-	-			
Enlarged lymph nodes	-				

AST: aspartate aminotransferase; ALT: alanine aminotransferase; BUN: blood urine nitrogen; CRP: C-reactive protein; LDH: lactate dehydrogenase.

### Molecular diagnosis

**Fig 2:**
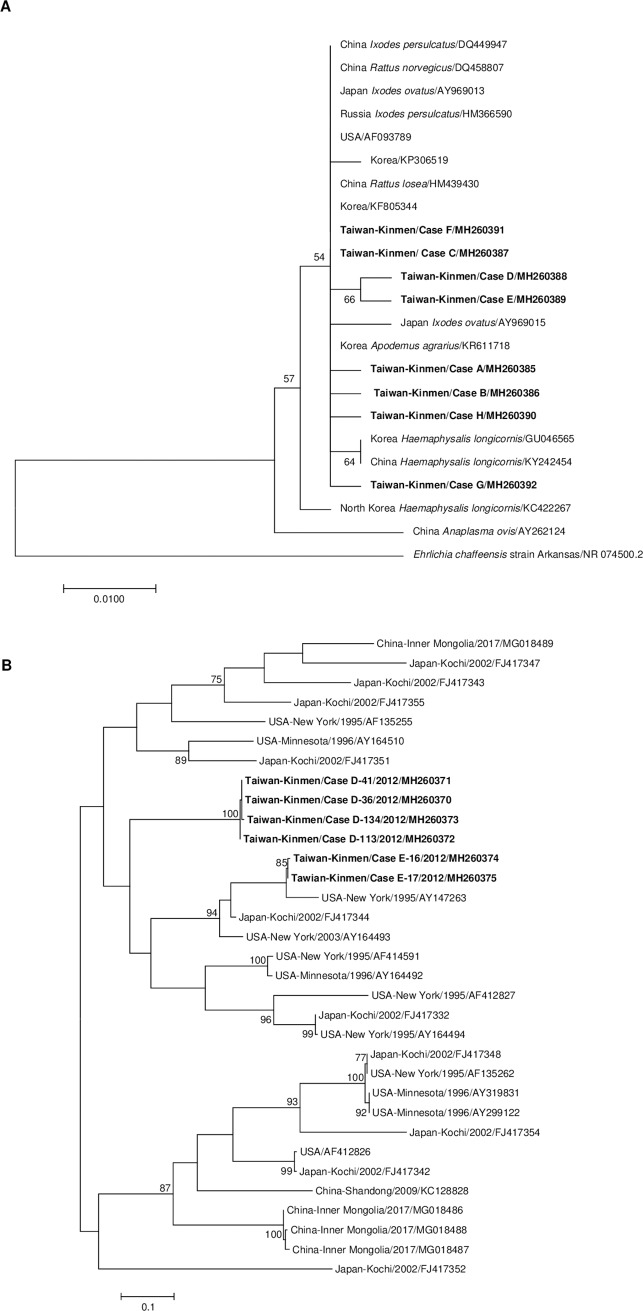
Phylogenetic analysis of *Anaplasma phagocytophilum* DNA sequences detected in human granulocytic anaplasmosis (HGA) patients in Kinmen, Taiwan, 2011–2012. **(A) The 16S rDNA.** Sequences derived from patients with HGA in Kinmen County in Taiwan (bold) were compared with *A*. *phagocytophilum* strains obtained in other countries. The evolutionary relationships was inferred by Maximum Likelihood method with 305 nucleotides. The percentage of trees was shown next to the branches. **(B) *p44/msp2* multigene family.** Amplicons of *p44/msp2* from two HGA patients in Kinmen were compared with other human isolates in the database. The tree was constructed using the Maximum Likelihood method. A total of 279 positions was involved in the final dataset, and the percentage higher than 75 was shown next to the branches. The GenBank accession numbers were indicated.

Of the 11 patients who tested positive for HGA serologically, 2 patients were confirmed by molecular diagnosis with evidence that both 16S rDNA and *p44/msp2* were successfully amplified. The evolutionary relationships was further inferred by molecular phylogenetic analysis for the 16S rDNA (Fig 2A, S2 Fig) and *p44/msp2* (Fig 2B, S3 Fig).

*Anaplasma phagocytophilum* 16S rDNA was detected in 8 patients (patients A-H) (Table 2). The resulting sequences that differed from each other by at least in 1 base, were submitted to GenBank (accession nos. MH260385-MH260392) (S2 Table). While two of the amplified fragments (from patients C and F) were identical to the reference sequence from Korea (accession no. MK271308.1), the others showed the highest degree of similarity to the sequences from Korea, the USA, Japan, and China (Fig 2A). The *p44/msp2* multigene was amplified in 2 patients (patients D and E). Subsequent cloning identified 4 different sequences from 85 clones from patient D (patient D-36, 41, 113, 134) and 2 sequences from 21 clones from patient E (patient E-16 and 17). All sequences were deposited in GenBank (accession nos. MH260370-MH260375) (S3 Table). Phylogenetic analysis revealed that the amplicons from the same patients clustered together, and the sequences were close to those of the variants identified in the USA and Japan (Fig 2B).

A 56-kDa TSA gene was detected in 4 patients (patients D-G). Further sequencing of the 56-kDa TSA gene showed that the PCR products in the study were identical to those of the isolates previously reported in Kinmen in 2006 (KM0606a, accession no. GQ332760; KM0605a, accession no. GQ332742; KM0607h, accession no. GQ332746) [34]. Patient D was infected with the Kawasaki strain of *O*. *tsutsugamushi* while the others were infected with the Karp strain. These strains of *O*. *tsutsugamushi* have continued to circulate in Kinmen County, where the habitat is favorable for chiggers and small mammals.

## Discussion

In this study, we reported granulocytic anaplasmosis in humans in Taiwan for the first time. Current or recent infection was suggested by seroconversion in paired serum samples from 11 patients. Molecular analysis confirmed *A*. *phagocytophilum* in 2 patients, and the amplified fragments shared high sequence similarity to the isolates from Korea, the USA, Japan, and China. Combined with the findings of previous studies that detected *A*. *phagocytophilum* DNA in small mammals and ticks, the transmission of the pathogen was further verified [23, 27]. Moreover, patients with concurrent HGA and scrub typhus were identified despite differences in Acari vectors, reflecting the unique ecosystem in Kinmen in which multiple pathogenic rickettsiae circulate. The Kinmen archipelago is nearly 200 km from the main island of Taiwan. With *A*. *phagocytophilum* DNA has been detected in animals on the main island of Taiwan, the scope of human infections requires further investigation [25, 26].

Although animal hosts and ticks have been reported to be infected by *A*. *phagocytophilum* worldwide, reports of infections in humans are less frequent, probably due to misdiagnosis owing to nonspecific clinical signs. Seroprevalence studies have shown that 14.9% of the residents in northwest Wisconsin, 17% of Slovenians, 2.6% of US military personnel, 16.2% of adults from western Norway, and 7.6% of adults in Yunnan Province in China have antibodies against *A*. *phagocytophilum* without a history suggestive of HGA [35–39]. This could imply the occurrence of subclinical infections. Nevertheless, a recent survey of hunters in eastern Poland detected seropositivity in 30% of the surveyed subjects, and more exposure was noted among those who handle animals than among blood donors from the general population in Belgium, suggesting that environment and animal contact history could be risk factors for infection [40, 41]. Serological evidence indicated that as many as 87 of the 274 subjects in this study had been exposed at some point to *A*. *phagocytophilum*, but no association was found between seropositivity and gender, occupation, or age. Because all participants presented with rickettsia-like fever upon enrollment, the at-risk population needs to be clarified by further reviewing the extent of *A*. *phagocytophilum* infection among all age groups of the general population.

None of the HGA patients recalled having recently experienced tick bites in the study. Similar findings have been observed, with at least 25% of patients with proven HGA failing to report exposure to ticks [1]. In addition, changes in the hematological and chemical blood tests of patients with HGA were nonspecific, in contrast with previous studies which showed that leukopenia, thrombocytopenia, and liver dysfunction were common in most HGA patients [21]. Nonetheless, serial measurements indicated that these abnormalities soon recovered after the first week of illness [42]. To further confirm *A*. *phagocytophilum* infection, PCR was performed with acute phase blood, and the 16S rDNA and *p44/msp2* were detected in 8 and 2 patients, respectively. Traditionally being used for screening tests, the 16S rDNA showed higher sensitivity in our findings despite its single copy in the pathogen perhaps due to the design of primers, shorter amplified fragments, specimen preservation or other reasons affecting PCR analysis and cloning. Specimens yielded positive results by both PCR were considered positive for molecular detection in current study. The resulting partial sequences of 16S rDNA were 99–100% identical to the reference sequence from Korea (accession no. MK271308.1) while the amplicons of *p44/msp2* were 92.5–100% identical to an isolate from the USA (accession no. CP006618.1). The conserved nature of the 16S rDNA and the more variable similarity of *p44/msp2* were in agreement with other report [43].

Kinmen has been recognized for its idyllic scenery and untouched ecology. During the Cold War era, the islands stood as the military frontier between the People’s Republic of China and Taiwan. The development of Kinmen was strictly focused on the ability to survive a long blockade. Drought-resistant sorghum was introduced for the production of liquor (kaoliang wine) as the major source of income. Agricultural and pastoral ways of life remained predominant on the islands until 1992, when tensions between mainland China and Taiwan gradually eased, and tourists began to visit across the strait. Today, the economy of Kinmen is mainly based on tourism. Investment and infrastructure projects have been undertaken, including the construction of houses, hotels, and businesses, in expectation of economic gains, but these changes also threaten characteristic local industries and traditional agricultural practices. An increase in the number of abandoned farms may have adverse consequences on the risk of disease and expose the residents not only to mite-borne scrub typhus but also tick-borne HGA [44].

Twenty-nine species of ticks belonging to the genera *Amblyomma*, *Aponomma*, *Boophilus*, *Dermacentor*, *Haemaphysalis*, *Ixodes*, and *Rhipicephalus* in the family Ixodidae have been documented in Taiwan [45]. Recent reports further recorded *Haemaphysalis lagrangei* parasitizing dogs and *Haemaphysalis wellingtoni*, *Ixodes columnae*, and *Ixodes turdus* parasitizing birds [46, 47]. While *I*. *persulcatus*, an important vector in northeast China, Russia, Japan, and Korea [10, 11, 48, 49], has not been encountered since 2000, studies from other countries demonstrated that *A*. *phagocytophilum* can infect the tick species that occur in Taiwan. The 16S rDNA from *A*. *phagocytophilum* has been detected in snake ticks (*Amblyomma helvolum* and *Aponomma varanense*) in Malaysia [50], *Amblyomma testudinarium* in Thailand and Japan [10, 51], *Rhipicephalus* (*Boophilus*) *microplus* in China [52], *Haemaphysalis formosensis* in Japan [10], *Ixodes nipponensis* in Korea [53], *Ixodes ovatus* in Japan [54], and *Ixodes simplex* in Hungary and Romania [55]. *Ixodes granulatus* and *R*. *haemaphysaloides* are the most common ticks collected from some small mammals captured in Kinmen County [56], and their infection with *A*. *phagocytophilum* has also been reported [23, 27], although the transmission cycle of *A*. *phagocytophilum* remains to be determined.

*Anaplasma phagocytophilum* infection can also be acquired via exposure to contaminated blood. Nosocomial infections have been reported in Anhui Province in China, suggesting that HGA can be acquired by contact with patient blood or respiratory secretions [57]. Similarly, infections have been reported in butchers exposed to infected deer blood [58]. Perinatal transmission was documented in 1 neonate [59]. A recent case of death from transfusion-transmitted anaplasmosis highlighted a new risk, as blood products are not currently screened for *A*. *phagocytophilum* infection [60]. In addition, *A*. *phagocytophilum* DNA was found in Tabanid flies, which could be potential vectors for transmission [61]. Whether these alternative routes play any roles in the presence of HGA in Taiwan should be explored.

Sequential or simultaneous infections of *A*. *phagocytophilum* with tick-borne pathogens such as *Borrelia burgdorferi*, *Babesia microti*, and *Rickettsia japonica* frequently occur after one or multiple tick bites [1, 10], but coinfection with mite-borne *O*. *tsutsugamushi* was never confirmed despite previous attempts in Korea [62, 63]. On the other hand, relatively high prevalence of *O*. *tsutsugamushi* infections in wild rodents, ranging from 69.1% to over 90%, as well as a high chigger infestation rate (100%, mostly *Leptotrombidium deliense*) and a high chigger *O*. *tsutsugamushi* PCR positivity rate (96%), have been found on the offshore islands and the main island of Taiwan [64–66]. Given that 15.8% to 17.2% of *R*. *losea*, the most abundant species in arable lands or abandoned fields in Kinmen, was infected by *A*. *phagocytophilum* and 19% parasitized by ticks [23, 25], coinfection is very likely to occur. The study employed IFA to detect antibodies of HGA and scrub typhus. Cross-reactive antibodies have been noted between *A*. *phagocytophilum* and *E*. *chaffeensis*, but cross-reactions between *A*. *phagocytophilum* and *B*. *burgdorferi* or *O*. *tsutsugamushi* were not significant in the previous studies [62, 67]. In our findings, concurrent positive reactions were observed in 8 among 129 patients with scrub typhus, and 4 of them had molecular evidence to support the diagnosis. Therefore, we concluded that the cross-reactions were not significant in the study and that patients simultaneously infected with *O*. *tsutsugamushi* and *A*. *phagocytophilum* were identified. In view of the similarity in the clinical presentations, infection or coinfection with other tick-borne pathogens, for example, *A*. *phagocytophilum*, should be considered for patients suspected of having scrub typhus in the future.

## Conclusions

We retrospectively examined blood samples from 274 patients with suspected diagnoses of scrub typhus in Kinmen in 2011 and 2012. IFA results showed that 87 patients (87/274; 31.8%) were seropositive for *A*. *phagocytophilum*, and 11 patients had evidence of seroconversion; that is, a 4-fold increase in the titer between acute and convalescent sera. Despite nonspecific clinical signs, active infection of *A*. *phagocytophilum* was confirmed by molecular diagnosis. Both of the 16S rDNA and *p44/msp2* gene were successfully amplified in 2 patients. Phylogenetic analysis revealed that the resulting sequences exhibited high similarity with the variants in Korea, the USA, Japan, and China. Our findings suggested HGA was present on the offshore island of Taiwan, and moreover, cases with concurrent HGA and scrub typhus were identified. *Anaplasma phagocytophilum* infection should be considered by the physicians for the purpose of early diagnosis and differential diagnosis in the area.

## Supporting information

S1 Checklist STROBE statement. (DOCX)

S1 Fig Monthly occurrence of patients with scrub typhus and human granulocytic anaplasmosis (HGA) in Kinmen County, 2011–2012. (JPG)

S2 Fig Phylogenetic analysis of *Anaplasma phagocytophilum* 16S rDNA sequences by Neighbor-Joining method. The associated taxa were clustered together in the bootstrap test (1000 replicates), and the percentage of replicate trees were shown next to the branches. A total of 305 nucleotides were analyzed in the final dataset.
(PPTX)

S3 Fig Phylogenetic analysis of *Anaplasma phagocytophilum p44/msp2* sequences by Neighbor-Joining method. The tree was constructed using the neighbor-joining method (bootstrap = 1000) with 279 nucleotides.
(PPTX)

S1 Table Clinical manifestations in the 11 patients with human granulocytic anaplasmosis (HGA) in Kinmen County, 2011–2012. (XLS)

S2 Table Comparison of *Anaplasma phagocytophilum* 16S rDNA partial sequence (305bp) from 8 cases of human granulocytic anaplasmosis (HGA) in Kinmen with the reference sequence from GenBank (accession number: KF805344). (DOCX)

S3 Table Comparison of *Anaplasma phagocytophilum p44/msp2* partial sequence from case D and case E with the reference sequence from GenBank (accession number: CP006618). (XLSX)

## References

[1] Bakken JS, Dumler JS. Human granulocytic anaplasmosis. Infect Dis Clin North Am. 2015;29(2):341–55. 10.1016/j.idc.2015.02.007 25999228 PMC4441757

[2] Blanco JR, Oteo JA. Human granulocytic ehrlichiosis in Europe. Clin Microbiol Infect. 2002;8(12):763–72. 12519349 10.1046/j.1469-0691.2002.00557.x

[3] Petrovec M, Lotric Furlan S, Zupanc TA,., Strle F, Brouqui P, Roux V, et al Human disease in Europe caused by a granulocytic *Ehrlichia* species. J Clin Microbiol. 1997;35(6):1556–9. 9163481 10.1128/jcm.35.6.1556-1559.1997 PMC229786

[4] Tylewska-Wierzbanowska S, Chmielewski T, Kondrusik M, Hermanowska-Szpakowicz T, Sawicki W, Sułek K. First cases of acute human granulocytic ehrlichiosis in Poland. Eur J Clin Microbiol Infect Dis. 2001;20(3):196–8. 10.1007/s100960100464 11347671

[5] Ruscio M, Cinco M. Human granulocytic ehrlichiosis in Italy: first report on two confirmed cases. Ann N Y Acad Sci. 2003;990:350–2. 10.1111/j.1749-6632.2003.tb07387.x 12860650

[6] Edouard S, Koebel C, Goehringer F, Socolovschi C, Jaulhac B, Raoult D, et al Emergence of human granulocytic anaplasmosis in France. Ticks Tick Borne Dis. 2012;3(5–6):403–5. 10.1016/j.ttbdis.2012.10.002 23182272

[7] Hagedorn P, Imhoff M, Fischer C, Domingo C, Niedrig M. Human granulocytic anaplasmosis acquired in Scotland, 2013. Emerg Infect Dis. 2014;20(6):1079–81. 10.3201/eid2006.131849 24857681 PMC4036789

[8] Gaowa Wulantuya, Yin X, Cao M, Guo S, Ding C, et al Case of human infection with *Anaplasma phagocytophilum* in Inner Mongolia, China. Jpn J Infect Dis. 2018;71(2):129–33. 10.7883/yoken.JJID.2017.301 29491236

[9] Zhang L, Wang G, Liu Q, Chen C, Li J, Long B, et al Molecular analysis of *Anaplasma phagocytophilum* isolated from patients with febrile diseases of unknown etiology in China. PLoS One. 2013;8(2):e57155 10.1371/journal.pone.0057155 23451170 PMC3579781

[10] Ohashi N, Gaowa, Wuritu, Kawamori F, Wu D, Yoshikawa Y, et al Human granulocytic anaplasmosis, Japan. Emerg Infect Dis. 2013;19(2):289–92. 10.3201/eid1902.120855 23460988 PMC3559047

[11] Kim KH, Yi J, Oh WS, Kim NH, Choi SJ, Choe PG, et al Human granulocytic anaplasmosis, South Korea, 2013. Emerg Infect Dis. 2014;20(10):1708–11. 10.3201/eid2010.131680 25271737 PMC4193166

[12] Lee SH, Park S, Lee YS, Lee HK, Hwang SD. Diagnosis and molecular characteristics of human infections caused by *Anaplasma phagocytophilum* in South Korea. J Microbiol. 2018;56(11):847–53. 10.1007/s12275-018-8385-8 30353471

[13] von Wissmann B, Hautmann W, Sing A, Hizo-Teufel C, Fingerle V. Assessing the risk of human granulocytic anaplasmosis and lyme borreliosis after a tick bite in Bavaria, Germany. Int J Med Microbiol. 2015;305(7):736–41. 10.1016/j.ijmm.2015.08.026 26338146

[14] Dugat T, Leblond A, Keck N, Lagrée AC, Desjardins I, Joulié A, et al One particular *Anaplasma phagocytophilum* ecotype infects cattle in the Camargue, France. Parasit Vectors. 2017;10(1):371 10.1186/s13071-017-2305-3 28764743 PMC5540577

[15] Narankhajid M, Yeruult C, Gurbadam A, Battsetseg J, Aberle SW, Bayartogtokh B, et al Some aspects on tick species in Mongolia and their potential role in the transmission of equine piroplasms, *Anaplasma phagocytophilum* and *Borrelia burgdorferi* L. Parasitol Res. 2018;117(11):3557–66. 10.1007/s00436-018-6053-x 30178195

[16] Foley J, Rejmanek D, Fleer K, Nieto N. Nidicolous ticks of small mammals in *Anaplasma phagocytophilum*-enzootic sites in northern California. Ticks Tick Borne Dis. 2011;2(2):75–80. 10.1016/j.ttbdis.2011.03.003 21686062 PMC3115731

[17] Herron MJ, Nelson CM, Larson J, Snapp KR, Kansas GS, Goodman JL. Intracellular parasitism by the human granulocytic ehrlichiosis bacterium through the P-selectin ligand, PSGL-. Science. 2000;288(5471):1653–6. 10.1126/science.288.5471.1653 10834846

[18] Park J, Choi KS, Dumler JS. Major surface protein 2 of *Anaplasma phagocytophilum* facilitates adherence to granulocytes. Infect Immun. 2003;71(7):4018–25. 10.1128/IAI.71.7.4018-4025.2003 12819090 PMC161989

[19] Garyu JW, Choi KS, Grab DJ, Dumler JS. Defective phagocytosis in A*naplasma phagocytophilum*-infected neutrophils. Infect Immun. 2005;73(2):1187–90. 10.1128/IAI.73.2.1187-1190.2005 15664962 PMC547103

[20] Ge Y, Yoshiie K, Kuribayashi F, Lin M, Rikihisa Y. *Anaplasma phagocytophilum* inhibits human neutrophil apoptosis via upregulation of bfl-1, maintenance of mitochondrial membrane potential and prevention of caspase 3 activation. Cell Microbiol. 2005;7(1):29–38. 10.1111/j.1462-5822.2004.00427.x 15617521

[21] Dumler JS, Choi KS, Garcia-Garcia JC, Barat NS, Scorpio DG, Garyu JW, et al Human granulocytic anaplasmosis and *Anaplasma phagocytophilum*. Emerg Infect Dis. 2005;11(12):1828–34. 10.3201/eid1112.050898 16485466 PMC3367650

[22] Dahlgren FS, Mandel EJ, Krebs JW, Massung RF, McQuiston JH. Increasing incidence of *Ehrlichia chaffeensis* and *Anaplasma phagocytophilum* in the United States, 2000–2007. Am J Trop Med Hyg. 2011;85(1):124–31. 10.4269/ajtmh.2011.10-0613 21734137 PMC3122356

[23] Weng MH, Tsai HP, Lin PR, Cheng KC, Guo MD, Lin CC. Surveillance of *Anaplasma phagocytophilum* infections in murines in Kinmen area, 2014. Taiwan Epidemiol Bull. 2015;31(14):347–55.

[24] Weng MH, Lien JC, Tsai HP, Lin PR, Cheng KC, Guo MD, et al Surveillance of *Anaplasma phagocytophilum* infection in rodents on Nangan island, Matsu. J Med Sci. 2013;33(5):279–84.

[25] Masuzawa T, Uchishima Y, Fukui T, Okamoto Y, Pan MJ, Kadosaka T, et al Detection of *Anaplasma phagocytophilum* and *Anaplasma bovis* in small wild mammals from Taichung and Kinmen Island, Taiwan. Jpn J Infect Dis. 2014;67(2):111–4. 24647253 10.7883/yoken.67.111

[26] Liu HJ, Yin CC, Hsieh YC, Chiang YC, Chang CD, Liao MH, et al Identification of the causative agents of *Ehrlichia canis* and *Anaplasma phagocytophilum* in dogs in Taiwan by nested PCR, indirect immunofluorescent-antibody assay, and sequence analysis of the 16S rRNA gene. Taiwan Vet J. 2006;32(2):76–87.

[27] Kuo CC, Huang JL, Chien CH, Shih HC, Wang HC. First molecular detection of *Anaplasma phagocytophilum* in the hard tick *Rhipicephalus haemaphysaloides* in Taiwan. Exp Appl Acarol. 2018;75:437–43. 10.1007/s10493-018-0283-6 30116923

[28] Moron CG, Popov VL, Feng HM, Wear D, Walker DH. Identification of the target cells of *Orientia tsutsugamushi* in human cases of scrub typhus. Mod Pathol. 2001;14(8):752–9. 10.1038/modpathol.3880385 11504834

[29] Taiwan National Infectious Disease Statistics System. Available online: https://nidss.cdc.gov.tw/en/ (accessed on 6 December 2018).

[30] Tsai KH, Chang SF, Yen TY, Shih WL, Chen WJ, Wang HC, et al Prevalence of antibodies against *Ehrlichia* spp. and *Orientia tsutsugamushi* in small mammals around harbors in Taiwan. Parasit Vectors. 2016;9:45 10.1186/s13071-016-1318-7 26817445 PMC4728797

[31] Parola P, Roux V, Camicas JL, Baradji I, Brouqui P, Raoult D. Detection of ehrlichiae in African ticks by polymerase chain reaction. Trans R Soc Trop Med Hyg. 2000;94(6):707–8. 10.1016/s0035-9203(00)90243-8 11198664

[32] Tsai KH, Lu HY, Tsai JJ, Yu SK, Huang JH, Shu PY. Human case of *Rickettsia felis* infection, Taiwan. Emerg Infect Dis. 2008;14(12):1970–2. 10.3201/eid1412.080515 19046543 PMC2634626

[33] Kumar S, Stecher 2, Tamura K. MEGA7: Molecular Evolutionary Genetics Analysis Version 7.0 for Bigger Datasets. Mol Biol Evol. 2016;33(7):1870–4. 10.1093/molbev/msw054 27004904 PMC8210823

[34] Lu HY, Tsai KH, Yu SK, Cheng CH, Yang JS, Su CL, et al Phylogenetic analysis of 56-kDa type-specific antigen gene of *Orientia tsutsugamushi* isolates in Taiwan. Am J Trop Med Hyg. 2010;83(3):658–63. 10.4269/ajtmh.2010.09-0608 20810835 PMC2929066

[35] Bakken JS, Goellner P, Van Etten M, Boyle DZ, Swonger OL, Mattson S, et al Seroprevalence of human granulocytic ehrlichiosis among permanent residents of northwestern Wisconsin. Clin Infect Dis. 1998;27(6):1491–6. 10.1086/515048 9868666

[36] Rojko T, Ursic T, Avsic-Zupanc T, Petrovec M, Strle F, Lotric-Furlan S. Seroprevalence of human anaplasmosis in slovene forestry workers. Ann N Y Acad Sci. 2006;1078:92–4. 10.1196/annals.1374.012 17114685

[37] Graf PC, Chretien JP, Ung L, Gaydos JC, Richards AL. Prevalence of seropositivity to spotted fever group rickettsiae and *Anaplasma phagocytophilum* in a large, demographically diverse US sample. Clin Infect Dis. 2008;46(4):70–7. 18171216 10.1086/524018

[38] Hjetland R, Henningsson AJ, Vainio K, Dudman SG, Grude N, Ulvestad E. Seroprevalence of antibodies to tick-borne encephalitis virus and *Anaplasma phagocytophilum* in healthy adults from western Norway. Infect Dis (Lond). 2015;47(1):52–6. 25342575 10.3109/00365548.2014.959044

[39] Wang F, Ma M, Luo S, Yan M, Tao L, Liu A, et al Seroprevalence of tick-borne *Anaplasma phagocytophilum* infection in healthy adult population and patients with acute undifferentiated fever from the Yunnan province of China. Vector Borne Zoonotic Dis. 2019:[Epub ahead of print]. 10.1089/vbz.2018.2389 30615589

[40] Tokarska-Rodak M, Plewik D, Michalski AJ, Kołodziej M, Mełgieś A, Pańczuk A, et al Serological surveillance of vector-borne and zoonotic diseases among hunters in eastern Poland. J Vector Borne Dis. 2016;53(4):355–61. 28035113

[41] De Keukeleire M, Vanwambeke SO, Cochez C, Heyman P, Fretin D, Deneys V, et al Seroprevalence of *Borrelia burgdorferi, Anaplasma phagocytophilum*, and *Francisella tularensis* infections in Belgium: results of three population-based samples. Vector Borne Zoonotic Dis. 2017;17(2):108–15. 10.1089/vbz.2016.1954 27828762

[42] Bakken JS, Aguero-Rosenfeld ME, Tilden RL, Wormser GP, Horowitz HW, Raffalli JT, et al Serial measurements of hematologic counts during the active phase of human granulocytic ehrlichiosis. Clin Infect Dis. 2001;32(6):862–70. 10.1086/319350 11247709

[43] Lee SH, Park S, Lee YS, Lee HK, Hwang SD. Diagnosis and molecular characteristics of human infections caused by *Anaplasma phagocytophilum* in South Korea. J Microbiol. 2018;56(11):847–853. 10.1007/s12275-018-8385-8 30353471

[44] Kuo CC, Huang JL, Shu PY, Lee PL, Kelt DA, Wang HC. Cascading effect of economic globalization on human risks of scrub typhus and tick-borne rickettsial diseases. Ecol Appl. 2012;22(6):1803–16. 10.1890/12-0031.1 23092017

[45] Robbins R. The ticks (Acari: Ixodida: Argasidae, Ixodidae)of Taiwan: a synonymic checklist. Proc Entomol Soc Wash. 2005;107(2):245–53.

[46] Chao LL, Hsieh CK, Ho TY, Shih CM. First zootiological survey of hard ticks (Acari: Ixodidae) infesting dogs in northern Taiwan. Exp Appl Acarol. 2019;77(1):105–15. 10.1007/s10493-018-0328-x 30488157

[47] Kuo CC, Lin YF, Yao CT, Shih HC, Chung LH, Liao HC, et al Tick-borne pathogens in ticks collected from birds in Taiwan. Parasit Vectors. 2017;10(1):587 10.1186/s13071-017-2535-4 29178908 PMC5702202

[48] Cao WC, Zhan L, He J, Foley JE, DE Vlas SJ, Wu XM, et al Natural *Anaplasma phagocytophilum* infection of ticks and rodents from a forest area of Jilin Province, China. Am J Trop Med Hyg. 2006;75(4):664–8. 17038691

[49] Rar VA, Epikhina TI, Yakimenko VV, Malkova MG, Tancev AK, Bondarenko EI, et al Genetic variability of *Anaplasma phagocytophilum* in ticks and voles from *Ixodes persulcatus*/*Ixodes trianguliceps* sympatric areas from Western Siberia, Russia. Ticks Tick Borne Dis. 2014;5(6):854–63. 10.1016/j.ttbdis.2014.07.008 25113979

[50] Kho KL, Koh FX, Tay ST. Molecular evidence of potential novel spotted fever group rickettsiae, *Anaplasma* and *Ehrlichia* species in *Amblyomma* ticks parasitizing wild snakes. Parasit Vectors. 2015;8:112 10.1186/s13071-015-0719-3 25889376 PMC4342797

[51] Nooroong P, Trinachartvanit W, Baimai V, Ahantarig A. Phylogenetic studies of bacteria (Rickettsia, Coxiella, and Anaplasma) in Amblyomma and Dermacentor ticks in Thailand and their co-infection. Ticks Tick Borne Dis. 2018;9(4):963–71. 10.1016/j.ttbdis.2018.03.027 29610046

[52] Zhang L, Liu H, Xu B, Lu Q, Li L, Chang L, et al *Anaplasma phagocytophilum* infection in domestic animals in ten provinces/cities of China. Am J Trop Med Hyg. 2012;87(1):185–9. 10.4269/ajtmh.2012.12-0005 22764312 PMC3391048

[53] Kang JG, Kim HC, Choi CY, Nam HY, Chae HY, Chong ST, et al Molecular detection of *Anaplasma*, *Bartonella*, and *Borrelia* species in ticks collected from migratory birds from Hong-do Island, Republic of Korea. Vector Borne Zoonotic Dis. 2013;13(4):215–25. 10.1089/vbz.2012.1149 23428091

[54] Wuritu Gaowa, Kawamori F, Aochi M, Masuda T, Ohashi N. Characterization of p44/msp2 multigene family of *Anaplasma phagocytophilum* from two different tick species, *Ixodes persulcatus* and *Ixodes ovatus*, in Japan. Jpn J Infect Dis. 2009;62(2):142–5. 19305056

[55] Hornok S, Szőke K, Meli ML, Sándor AD, Görföl T, Estók P, et al Molecular detection of vector-borne bacteria in bat ticks (Acari: Ixodidae, Argasidae) from eight countries of the Old and New Worlds. Parasit Vectors. 2019;12(1):50 10.1186/s13071-019-3303-4 30670048 PMC6343265

[56] Tsui PY, Tsai KH, Weng MH, Hung YW, Liu YT, Hu KY, et al Molecular detection and characterization of spotted fevergroup rickettsiae in Taiwan. Am J Trop Med Hyg. 2007;77(5):883–90. 17984347

[57] Zhang L, Liu Y, Ni D, Li Q, Yu Y, Yu XJ, et al Nosocomial transmission of human granulocytic anaplasmosis in China. JAMA. 2008;300(19):2263–70. 10.1001/jama.2008.626 19017912

[58] Bakken JS, Krueth JK, Lund T, Malkovitch D, Asanovich K, Dumler JS. Exposure to deer blood may be a cause of human granulocytic ehrlichiosis. Clin Infect Dis. 1996;23(1):198 10.1093/clinids/23.1.198 8816164

[59] Horowitz HW, Kilchevsky E, Haber S, Aguero-Rosenfeld M, Kranwinkel R, James EK, et al Perinatal transmission of the agent of human granulocytic ehrlichiosis. N Engl J Med. 1998;339(6):375–8. 10.1056/NEJM199808063390604 9691104

[60] Goel R, Westblade LF, Kessler DA, Sfeir M, Slavinski S, Backenson B, et al Death from transfusion-transmitted anaplasmosis, New York, USA, 2017. Emerg Infect Dis. 2018;24(8):1548–50. 10.3201/eid2408.172048 30016241 PMC6056119

[61] Werszko J, Szewczyk T, Steiner-Bogdaszewska Z, Laskowski Z, Karbowiak G. Molecular detection of *Anaplasma phagocytophilum* in blood-sucking flies (Diptera: Tabanidae) in Poland. J Med Entomol. 2019. 10.1093/jme/tjy217 30615168

[62] Park JH, Heo EJ, Choi KS, Dumler JS, Chae JS. Detection of antibodies to *Anaplasma phagocytophilum* and *Ehrlichia chaffeensis* antigens in sera of Korean patients by western immunoblotting and indirect immunofluorescence assays. Clin Diagn Lab Immunol. 2003;10(6):1059–64. 10.1128/CDLI.10.6.1059-1064.2003 14607867 PMC262439

[63] You MJ, Kim WI, Cho HS, Shin GW, Hwang JH, Lee CS. Human anaplasmosis in acute febrile patients during scrub typhus season in Korea. Infect Chemother. 2015;47(3):181–2. 10.3947/ic.2015.47.3.181 26483992 PMC4607771

[64] Wang HC, Chung CL, Lin TH, Wang CH, Wu WJ. Studies on the vectors and pathogens of scrub typhus on murine-like animals in Kinmen County, Taiwan. Formosa Entomol. 2004;24:257–72.

[65] Lin PR, Tsai HP, Weng MH, Lin HC, Chen KC, Kuo MD, et al Field assessment of *Orientia tsutsugamushi* infection in small mammals and its association with the occurrence of human scrub typhus in Taiwan. Acta Trop. 2014;131:117–23. 10.1016/j.actatropica.2013.11.029 24361181

[66] Kuo CC, Lee PL, Chen CH, Wang HC. Surveillance of potential hosts and vectors of scrub typhus in Taiwan. Parasit Vectors. 2015;8:611 10.1186/s13071-015-1221-7 26626287 PMC4666075

[67] Bunnell JE, Magnarelli LA, Dumler JS. Infection of laboratory mice with the human granulocytic ehrlichiosis agent does not induce antibodies to diagnostically significant *Borrelia burgdorferi* antigens. J Clin Microbiol. 1999;37(6):2077–9. 10325386 10.1128/jcm.37.6.2077-2079.1999 PMC85039

