# Supplementary figures and images for: Human granulocytic anaplasmosis in Kinmen, an offshore island of Taiwan
Source: PLoS Negl Trop Dis. 2019 Sep 20;13(9):e0007728. doi: 10.1371/journal.pntd.0007728 (PMC6774531; doi:10.1371/journal.pntd.0007728)

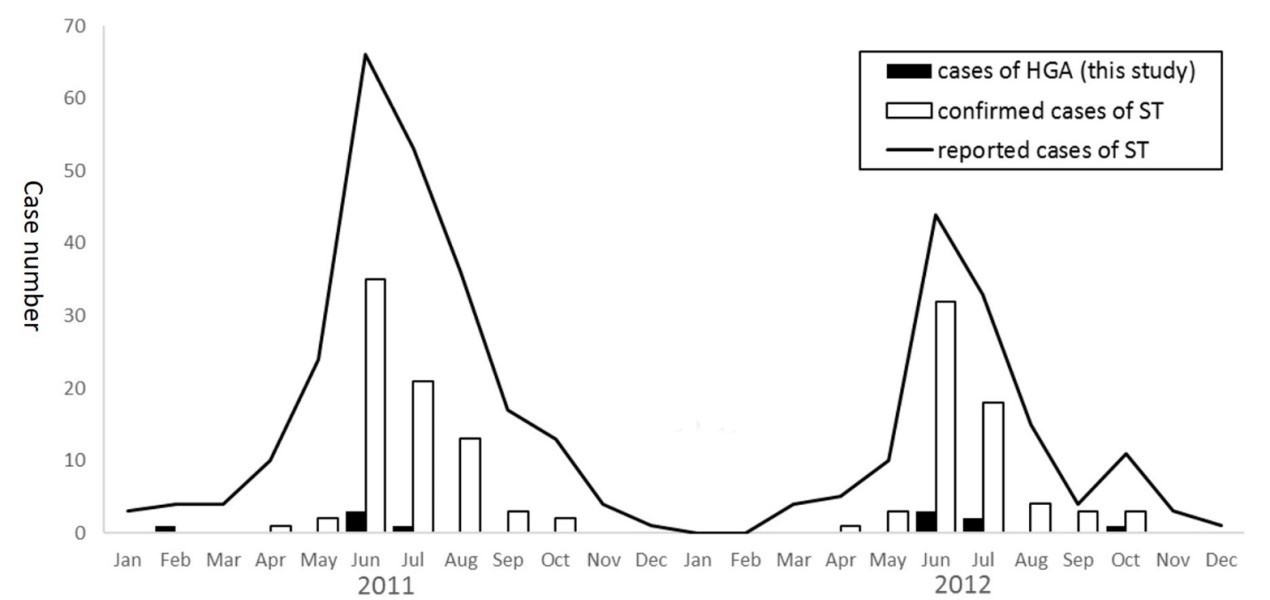

Supplement: S1 Fig — (JPG) [file pntd.0007728.s002.jpg]
