# Supplementary material for: Human granulocytic anaplasmosis in Kinmen, an offshore island of Taiwan
Source: PLoS Negl Trop Dis. 2019 Sep 20;13(9):e0007728. doi: 10.1371/journal.pntd.0007728 (PMC6774531; doi:10.1371/journal.pntd.0007728)
Supplement: S2 Fig — The associated taxa were clustered together in the bootstrap test (1000 replicates), and the percentage of replicate trees were shown next to the branches. A total of 305 nucleotides were analyzed in the final dataset. (PPTX) [file pntd.0007728.s003.pptx]

## Slide 1
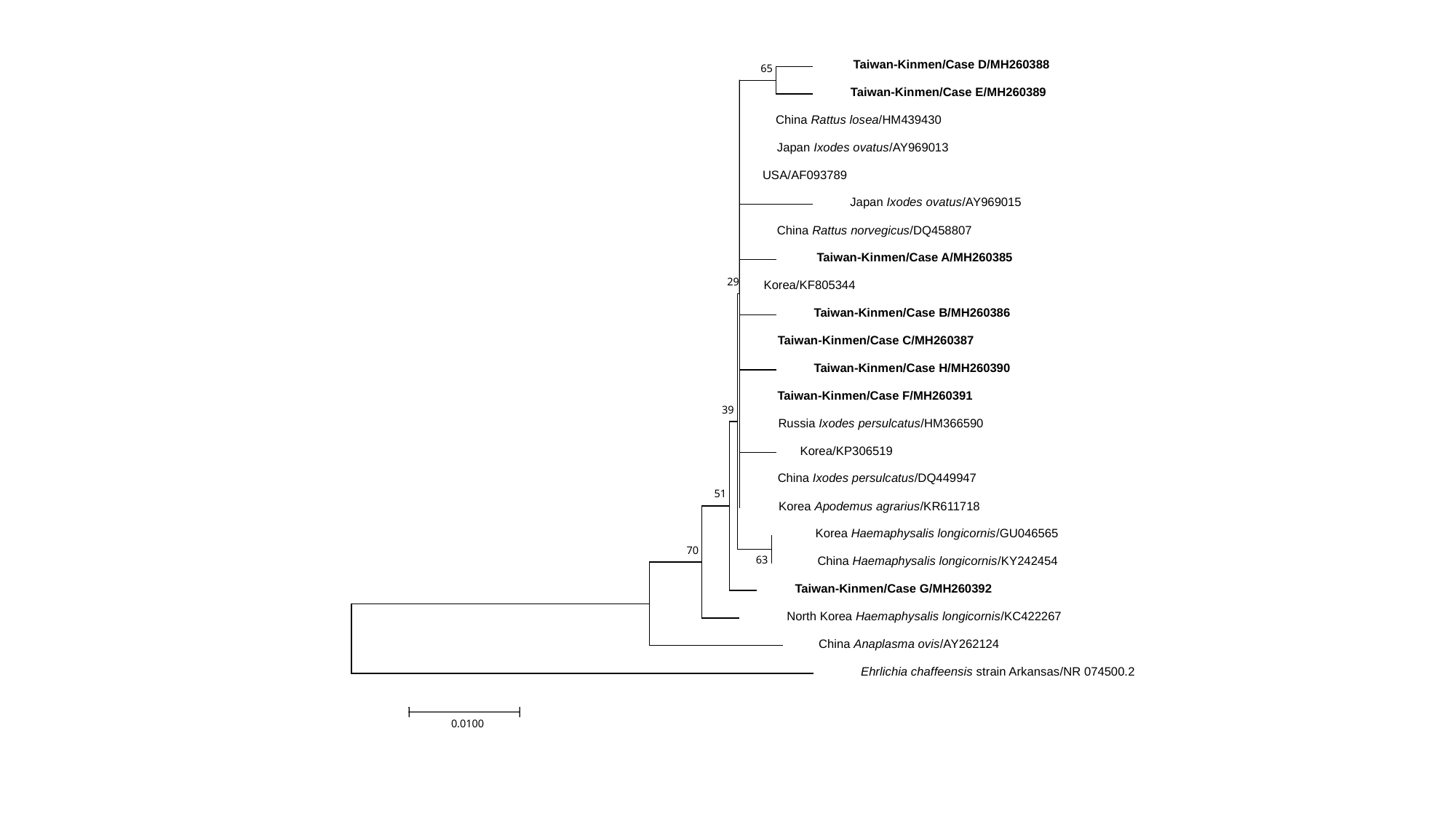

Taiwan-Kinmen/Case D/MH260388
65
 Taiwan-Kinmen/Case E/MH260389
 China Rattus losea/HM439430
 Japan Ixodes ovatus/AY969013
 USA/AF093789
 Japan Ixodes ovatus/AY969015
 China Rattus norvegicus/DQ458807
 Taiwan-Kinmen/Case A/MH260385
29
 Korea/KF805344
 Taiwan-Kinmen/Case B/MH260386
 Taiwan-Kinmen/Case C/MH260387
 Taiwan-Kinmen/Case H/MH260390
 Taiwan-Kinmen/Case F/MH260391
39
 Russia Ixodes persulcatus/HM366590
 Korea/KP306519
 China Ixodes persulcatus/DQ449947
51
 Korea Apodemus agrarius/KR611718
 Korea Haemaphysalis longicornis/GU046565
70
63
 China Haemaphysalis longicornis/KY242454
 Taiwan-Kinmen/Case G/MH260392
 North Korea Haemaphysalis longicornis/KC422267
 China Anaplasma ovis/AY262124
 Ehrlichia chaffeensis strain Arkansas/NR 074500.2
0.0100
