# Supplementary material for: Human granulocytic anaplasmosis in Kinmen, an offshore island of Taiwan
Source: PLoS Negl Trop Dis. 2019 Sep 20;13(9):e0007728. doi: 10.1371/journal.pntd.0007728 (PMC6774531; doi:10.1371/journal.pntd.0007728)
Supplement: S3 Fig — The tree was constructed using the neighbor-joining method (bootstrap = 1000) with 279 nucleotides. (PPTX) [file pntd.0007728.s004.pptx]

## Slide 1
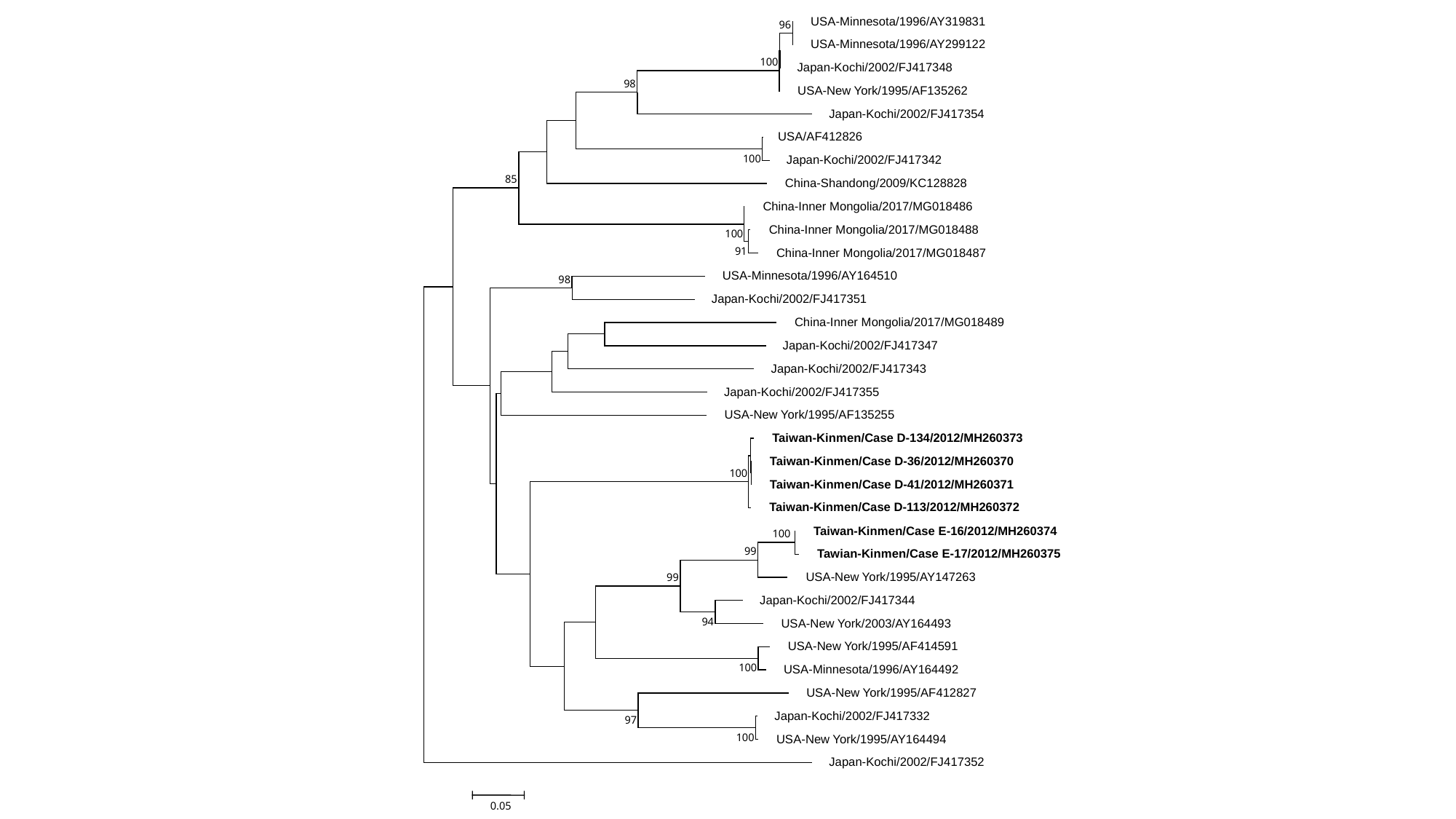

USA-Minnesota/1996/AY319831
96
 USA-Minnesota/1996/AY299122
100
 Japan-Kochi/2002/FJ417348
98
 USA-New York/1995/AF135262
 Japan-Kochi/2002/FJ417354
 USA/AF412826
100
 Japan-Kochi/2002/FJ417342
85
 China-Shandong/2009/KC128828
 China-Inner Mongolia/2017/MG018486
 China-Inner Mongolia/2017/MG018488
100
91
 China-Inner Mongolia/2017/MG018487
 USA-Minnesota/1996/AY164510
98
 Japan-Kochi/2002/FJ417351
 China-Inner Mongolia/2017/MG018489
 Japan-Kochi/2002/FJ417347
 Japan-Kochi/2002/FJ417343
 Japan-Kochi/2002/FJ417355
 USA-New York/1995/AF135255
 Taiwan-Kinmen/Case D-134/2012/MH260373
 Taiwan-Kinmen/Case D-36/2012/MH260370
100
 Taiwan-Kinmen/Case D-41/2012/MH260371
 Taiwan-Kinmen/Case D-113/2012/MH260372
 Taiwan-Kinmen/Case E-16/2012/MH260374
100
99
 Tawian-Kinmen/Case E-17/2012/MH260375
 USA-New York/1995/AY147263
99
 Japan-Kochi/2002/FJ417344
94
 USA-New York/2003/AY164493
 USA-New York/1995/AF414591
100
 USA-Minnesota/1996/AY164492
 USA-New York/1995/AF412827
 Japan-Kochi/2002/FJ417332
97
100
 USA-New York/1995/AY164494
 Japan-Kochi/2002/FJ417352
0.05
