# Supplementary material for: Human granulocytic anaplasmosis in Kinmen, an offshore island of Taiwan
Source: PLoS Negl Trop Dis. 2019 Sep 20;13(9):e0007728. doi: 10.1371/journal.pntd.0007728 (PMC6774531; doi:10.1371/journal.pntd.0007728)
Supplement: S2 Table — (DOCX) [file pntd.0007728.s006.docx]

**S2 Table. Comparison of *Anaplasma phagocytophilum* 16S rDNA partial sequence (305bp) from 8 cases of human granulocytic anaplasmosis (HGA) in Kinmen with the reference sequence from GenBank (accession number: KF805344)**

| ***Anaplasma phagocytophilum*** 16S rDNA partial sequence (305bp) | | | | | | | |
| --- | --- | --- | --- | --- | --- | --- | --- |
| KF805344 | 139(A) | 196(C) | 252(G) | 254(T) | 264(A) | 265(A) | 287(A) |
| case A (MH260385) | . | 196(T) | . | . | . | . | . |
| case B (MH 260386) | . | . | . | . | . | . | 287(G) |
| case C (MH 260387) | . | . | . | . | . | . | . |
| case D (MH260388) | 139(G) | . | . | . | . | 265(G) | . |
| case E (MH260389) | . | . | . | 254(C) | . | 265(G) | . |
| case F (MH260391) | . | . | . | . | . | . | . |
| case G (MH260392) | . | . | 252(A) | . | . | . | . |
| case H (MH260390) | . | . | . | . | 264(G) | . | . |
